# Direct Communication Between Brains: A Systematic PRISMA Review of Brain-To-Brain Interface

**DOI:** 10.3389/fnbot.2021.656943

**Published:** 2021-05-07

**Authors:** Chang S. Nam, Zachary Traylor, Mengyue Chen, Xiaoning Jiang, Wuwei Feng, Pratik Yashvant Chhatbar

**Affiliations:** ^1^Edward P. Fitts Department of Industrial & Systems Engineering, North Carolina State University, Raleigh, NC, United States; ^2^Department of Electrical & Computer Engineering, North Carolina State University, Raleigh, NC, United States; ^3^Department of Neurology, Duke University, Durham, NC, United States

**Keywords:** brain-to-brain interface, brain-computer interface, computer-brain interface, brain communication, neuroergonomics

## Abstract

This paper aims to review the current state of brain-to-brain interface (B2BI) technology and its potential. B2BIs function via a brain-computer interface (BCI) to read a sender's brain activity and a computer-brain interface (CBI) to write a pattern to a receiving brain, transmitting information. We used the Preferred Reporting Items for Systematic Reviews and Meta-Analyses (PRISMA) to systematically review current literature related to B2BI, resulting in 15 relevant publications. Experimental papers primarily used transcranial magnetic stimulation (tMS) for the CBI portion of their B2BI. Most targeted the visual cortex to produce phosphenes. In terms of study design, 73.3% (11) are unidirectional and 86.7% (13) use only a 1:1 collaboration model (subject to subject). Limitations are apparent, as the CBI method varied greatly between studies indicating no agreed upon neurostimulatory method for transmitting information. Furthermore, only 12.4% (2) studies are more complicated than a 1:1 model and few researchers studied direct bidirectional B2BI. These studies show B2BI can offer advances in human communication and collaboration, but more design and experiments are needed to prove potential. B2BIs may allow rehabilitation therapists to pass information mentally, activating a patient's brain to aid in stroke recovery and adding more complex bidirectionality may allow for increased behavioral synchronization between users. The field is very young, but applications of B2BI technology to neuroergonomics and human factors engineering clearly warrant more research.

## Introduction

In the past decade or so, a new neural interface technology, also known as a brain-to-brain interface (B2BI), has entered literature as an extension of the usual applications of neuroimaging technology, measuring one's brain activity such as brain-computer interface (BCI, Nam et al., 2018), and brain stimulation technology, activating the brain directly with electricity (hereinafter computer-brain interface or CBI), to a multi-subject (sender-receiver) approach. B2BI allows two brains to mutually exchange decoded neural information with each other through a BCI that reads a sender's brain activity and a CBI that writes the delivered brain activity to a receiving brain.

Since its proof of concept by Pais-Vieira et al. (2013), B2BI has found several interesting applications, ranging from simply transmitting binary information (Grau et al., 2014) to creating biological neural networks (Pais-Vieira et al., 2015). Perhaps more excitingly, B2BIs have been used to issue instructions to users (Jiang et al., 2019) and respond to questions (Stocco et al., 2015). While these applications still only apply binary information transfer, they show more complex applications of such communication. Mashat et al. (2017) created a B2BI system more focused on rehabilitation for patients. By combining a B2BI with functional electrical stimulation (FES), they argued that systems like this could allow more advanced physical therapy. Lee et al. (2017) argued that brain-to-brain systems could eventually be applied to create thought-based communication between people and even closed-loop feedback of one's own brain activity.

**Figure 1:**
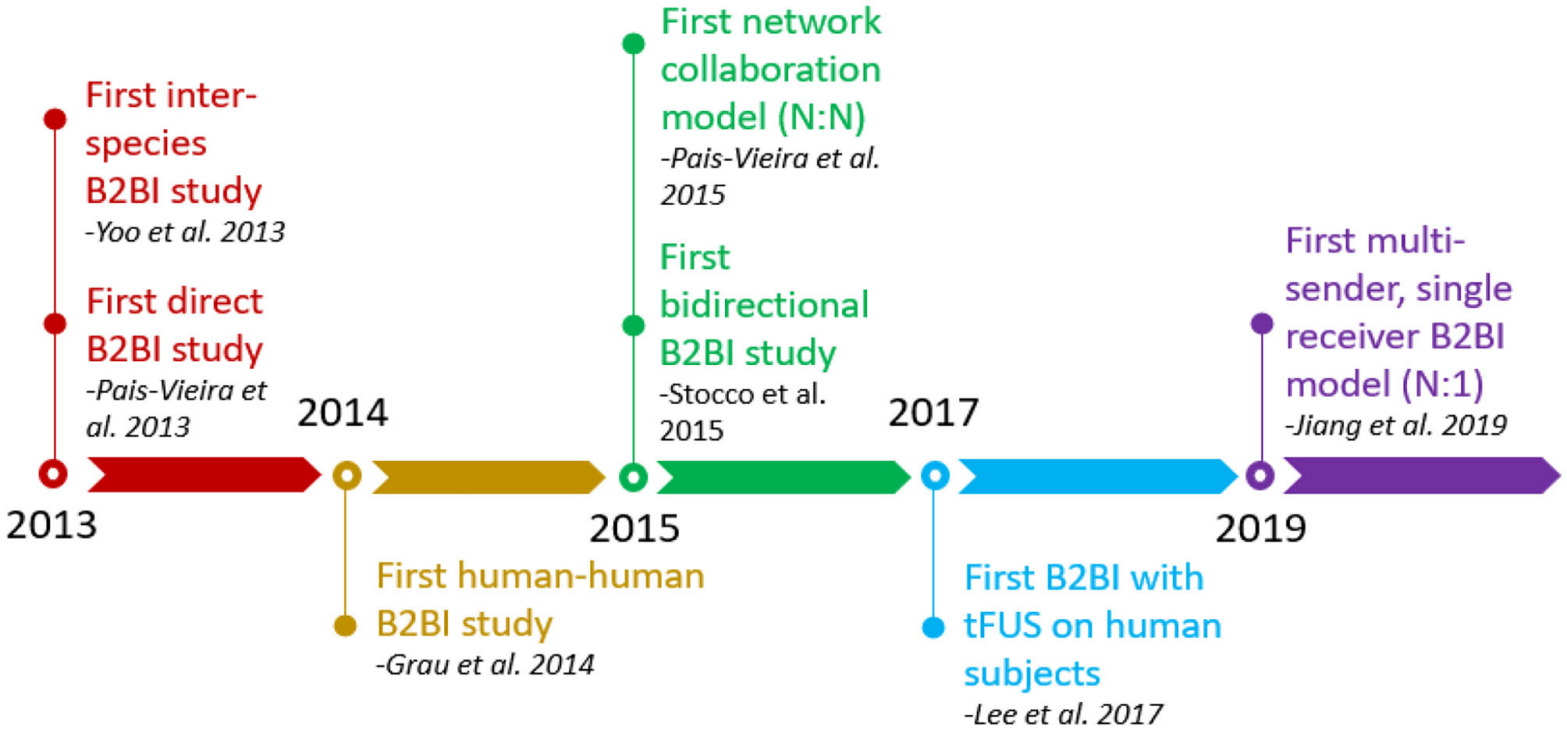
Timelines of advancement in brain-to-brain interface (B2BI) research.

Figure 1 shows a timeline of major events in B2BI research. Though the first direct B2BI study involving transmitting sensorimotor information between rodents was conducted in 2013, there existed very simple proof of concept studies and exploratory literature on the subject as early as 2011. Early studies used rodent models to test their devices, with Yoo et al. (2013) controlling a rodent via a human connected to EEG, but the first human-to-human study arrived from Grau et al. (2014). Stocco et al. (2015) were the first to employ a bidirectional design, transmitting information via magnetic stimulation in one direction and visual feedback in the other. The same year, Pais-Vieira expanded on their earlier system to create a biological neural network through multiple ICM rats connected bidirectionally. Stocco's system was expanded on by Jiang et al. (2019) with a different task, expanding the indirect bidirectional B2BI literature.

### Research Motivation

Despite the potential of its applications, B2BI is still in its infancy and has a long way to go before mainstream adoption. In particular, the contemporary B2BI research calls for additional investigations in order to progress to maturity. First, no systematic review study has been conducted in the field; to the best of our knowledge, no comprehensive review study of any kind has been conducted. This has the effect of isolating studies from each other rather than forming a complete body of research. Alongside the advances made in the field, B2BI research also identifies many gaps in the literature that warrant further investigation. Though small in number, the currently published B2BI studies pose various problems, including (i) how to highlight methodological concerns in research studies (Eagly and Wood, 1994) critical in improving future work and (ii) how to identify questions and areas where further research is or is not necessary (Mahood et al., 2014). A systematic review, defined as a “review of the evidence on a clearly formulated question that uses systematic and explicit methods to identify” (Jahan et al., 2016, p. 1), address both of these issues. A systematic review selects and analyzes relevant research to extract and analyze the data present. To our knowledge, existing literature on B2BI has not been reviewed in depth, and we present the first systematic review of the field. In this study, we seek to provide a systematic review of B2BI literature allowing researchers, both current and future, the ability to understand the state-of-the-art of B2BI as well as determine future directions, topics, and terminology of the field.

**Figure 2:**
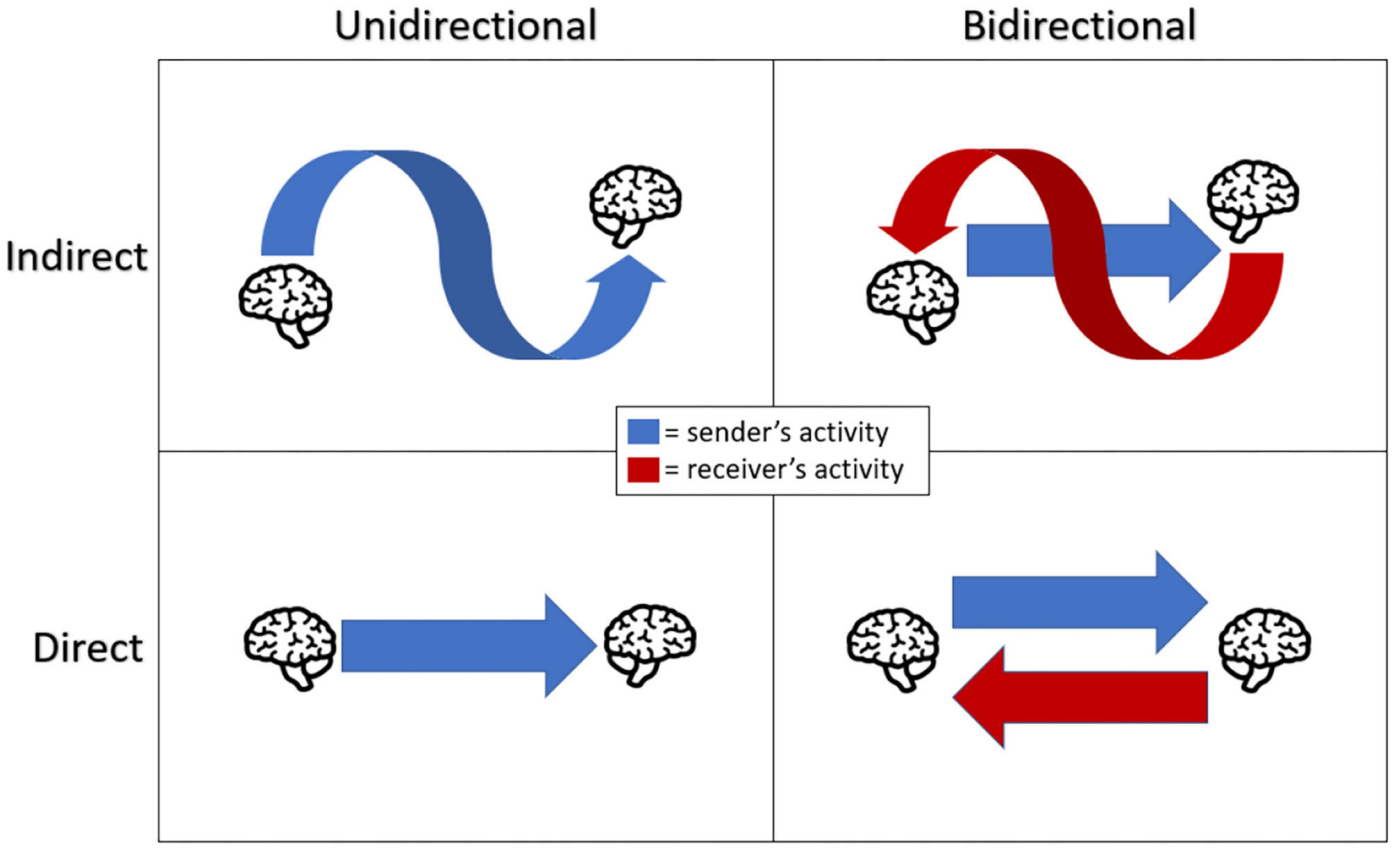
A graphical scheme of collaboration type. Directionality refers to the flow of information, either just from sender to receiver or in both directions. Directness refers to the use of brain stimulation to send information (a straight arrow) or other means (a curved arrow). It is important to note that indirect unidirectional systems do not qualify as B2Bs by our definition and that indirect bidirectional systems could involve indirect (curved arrow) information transmission from the sender or the receiver, not just as depicted.

In regards to that final point, the exact definition of a B2BI varies between publications, and no set definition is agreed upon or standardized. From the literature, B2BI can be defined as a system, composed of a BCI and CBI portion, which records a user's brain activity and uses it to modulate another user's brain activity allowing information transfer between the two brains. However, this definition does not accurately reflect the rich diversity of B2BI systems. Through this systematic review, we will define current B2Bs in terms of directionality (the flow of information) and directness (the use of brain stimulation to send information) (see Figure 2); in this study a true B2BI is defined as a direct bidirectional B2BI. Unidirectional systems only transmit information from one subject to another, while bidirectional systems allow for the transmission of information back in a call-and-response design. Each of these directions can also be labeled as direct or indirect, indicating the means through which information is sent. Direct transmission involves activation of the receiving brain via the B2BI, through some means such as magnetic, ultrasonic, or electrical stimulation. Direct B2BIs employ only neuromodulation to impart information to the receiver. Indirect transmission refers to any system that uses any method other than neuromodulation at any point of the communication. Direct and indirect uni and bidirectional B2BIs, though fundamentally different in their design, often have similar applications. B2BIs show potential in allowing communication with locked-in patients, advanced user state monitoring, and even potential military applications (Hildt, 2019). Some literature that discusses B2BI does not meet these criteria; research by Maksimenko et al. (2018) is fascinating and adjacent to the field, but we believe closer relates to the subject of hyperscanning and is outside the scope of this review. James (2011) has a similar issue, discussing and theorizing about brain-to-brain communication however failing to present a device that we think qualifies as a true B2BI. These definitions allow us, as well as other researchers, to continue to analyze and produce research in this new field. Finally, we seek to identify research issues that have not been fully addressed by the literature to date. Establishing research directions and questions can help guide researchers looking for new avenues and investigations to pursue in the budding field of B2BI. In particular, this study focused on four main research questions (RQs) regarding (1) BCI methodology, (2) CBI methodology, (3) Collaboration type, and (4) Collaboration model by subject type.

Chronologically, B2BI research has advanced from indirect unidirectional systems to direct unidirectional systems to indirect bidirectional systems. Early devices presented by James in 2011, as well as other indirect unidirectional systems, bear most resemblance to traditional BCI systems with visual feedback rather than actual B2BIs. It was not until 2013–2015 that direct B2BI systems became commonplace in research. From there, researchers began to improve the systems and develop new paradigms to test their applications. Such improvements include use of transcranial focused ultrasonic stimulation (tFUS) for increased spatial accuracy of neuromodulation (Lee et al., 2017) in non-invasive B2BI. Rodent studies are often capable of invasive methods of neurostimulation, and thus research in the animal model began with technology like implanted electrodes (Pais-Vieira et al., 2013; Yu et al., 2014) and have equally advanced into technology such as optogenetic recording and stimulation of rat brains (Lu et al., 2020). Beyond just the individual BCI and CBI devices or methods employed, task design has evolved as well. Referred to in this paper as the collaboration model, most studies in this review employ a 1:1 model, however N:N models involving transmission of information between a network of rodent brains have been tested (Pais-Vieira et al., 2015). In 2019, Jiang et al. added to the body of literature supporting the collaborative potential of B2BI with a N:1 collaboration model.

### Review Objectives

The overarching objective of this study was to conduct a comprehensive review on B2BI research, with the goal of systematically identifying, critically appraising, and synthesizing all relevant studies on neural communication between two or more brains. An explicit systematic method, the Preferred Reporting Items for Systematic Reviews and Meta-Analyses (PRISMA) was used to address four specific research questions (RQs) regarding BCI methodology (RQ1), CBI methodology (RQ2), collaboration type (RQ3), and collaboration model by subject type (RQ4) which together articulate the current-state-of research being conducted in B2BI. PRISMA is known to minimize bias and thus provide reliable findings from which conclusions can be drawn (Liberati et al., 2009). To the best of our knowledge, this is the first systematic review study that used PRISMA to compile all relevant and cutting-edge B2BI research to address the current state-of-the-art brain-to-brain interface research since its first publication in 2011.

**RQ1. What is the BCI system employed and what region of the brain is neural activity recorded?** To answer this BCI methodology-related question, we analyze the usage frequency of different BCI methods in the 15 selected research papers on B2BI. Due to the small body of literature, all 15 studies can be divided into using either electroencephalogram (EEG) recording, intracortical microelectrodes (ICM), or optogenetics. For increased clarity, these categories were further subdivided into the region of the brain that the methods targeted: the motor cortex, somatosensory cortex, visual cortex, or the nucleus incertus. The BCI method used is important, as it relates to not only the task performed by the “sender” in a B2BI system, but also the type of information being encoded and sent (i.e., motor movements).

**RQ2. What is the CBI system employed and what method does that system use to elicit neural activity?** To address the CBI methodology of B2BI systems in literature, this paper analyzes the usage frequency of different CBI technology and the regions of the brain that they target. B2BI studies utilize either intracortical microelectrodes (ICM), transcranial magnetic stimulation (tMS), transcranial focused ultrasonic stimulation (tFUS), or optogenetics. These devices were used to stimulate either somatosensory cortex, motor cortex, or visual cortex (phosphenes) in humans. In animal models, either the somatosensory cortex, nigrostriatal pathway, nucleus incertus, or antenna are targeted. The CBI methodology indicates what the “receiver” is intended to do in a given B2BI task (i.e., move left or right).

**RQ3. Which of the four categories (indirect/direct unidirectional or indirect/direct bidirectional) does the collaboration between subjects fall under?** To address the question of collaboration type, we categorize all of these selected literature based on directionality. As mentioned previously, these terms refer to the overall design of the B2BI system, indicating how the participants were able to communicate with each other (i.e., using peripheral nervous system pathways or direct neuromodulation).

**RQ4. Through what model (1:1, N:1, 1:N, N:N) do the subjects collaborate?** To answer this collaboration model-related question, we also further divide papers by species of subject, as several studies employ cross-species B2BI. The 15 experimental research papers selected only utilize humans, rodents, and cockroaches as subjects. Inter-species pairs exist on several occasions, and answering this question allows us to determine the application of B2BI systems (e.g., communication, team collaboration, decision making).

## Review Method

We applied the systematic approach PRISMA (Liberati et al., 2009) in this review. Research articles were gathered from four different databases: (a) IEEE Xplore for a technology perspective; (b) PubMed, for a medical perspective, (c) Engineering Village, for an engineering perspective; and (d) Web of Science for a cross-disciplinary perspective (Powers et al., 2015).

### Inclusion and Prescreening Criteria

Inclusion criteria were English articles written between 2013 and August 18th 2020. The first experiment conducted using direct B2BI was published in 2013, so that year functioned as our starting point. Unpublished or working papers, dissertations, news articles, book chapters, conference papers, and ethical reviews were excluded. Experimental research will be the focus of this analysis, but mention will be given to those papers that do not conduct an experiment but still contribute information to the budding field of brain-to-brain communication.

**Figure 3:**
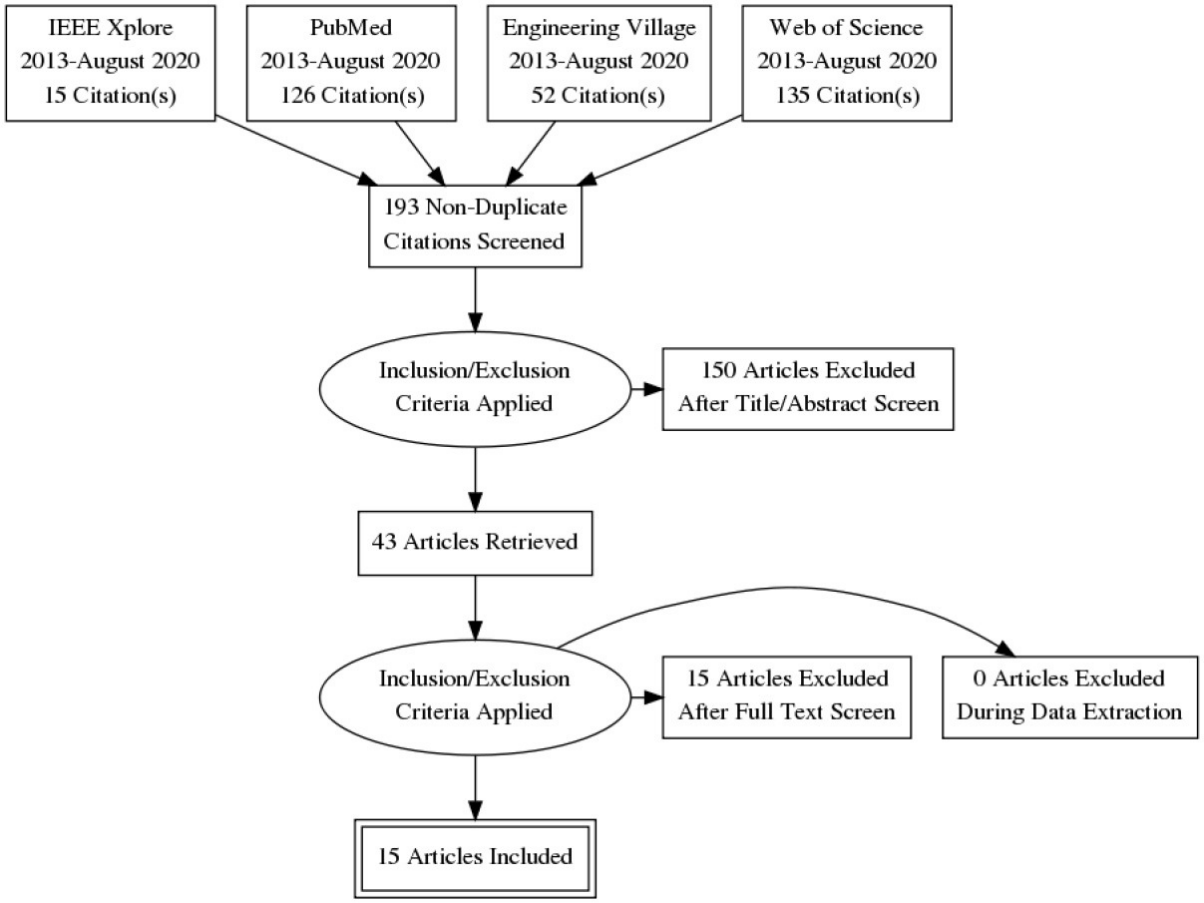
PRISMA flow diagram of brain-to-brain interface research paper review.

The search term used in all four search engines was “brain to brain.” Typically, a systematic review might include a more complex search term, however there are very few publications on this subject and even fewer domains that the technology has been applied to. Figure 3 shows the flow diagram of PRISMA with the number of studies from each online database. After the keyword search, duplicates were removed and 193 articles remained. Those articles were screened again based on titles and abstracts, and 43 studies remained. Lastly, 15 experiment-conducting articles were selected.

### Eligibility Criteria

This review pertains specifically to experiments conducted with B2BI devices. Experiment studies where subjects' brain activities were measured simultaneously but no data was actively “sent” from one brain to the other were excluded. Due to the small pool of literature, further screening based on subject or experimental design (control group, measured variables, etc.) proved infeasible. The main question when screening full text articles for eligibility was whether or not the study recorded activation from one brain that was used to selectively modulate activation in a different brain. Screening based on this question resulted in the 15 selected experimental studies.

## Results

**Figure 4:**
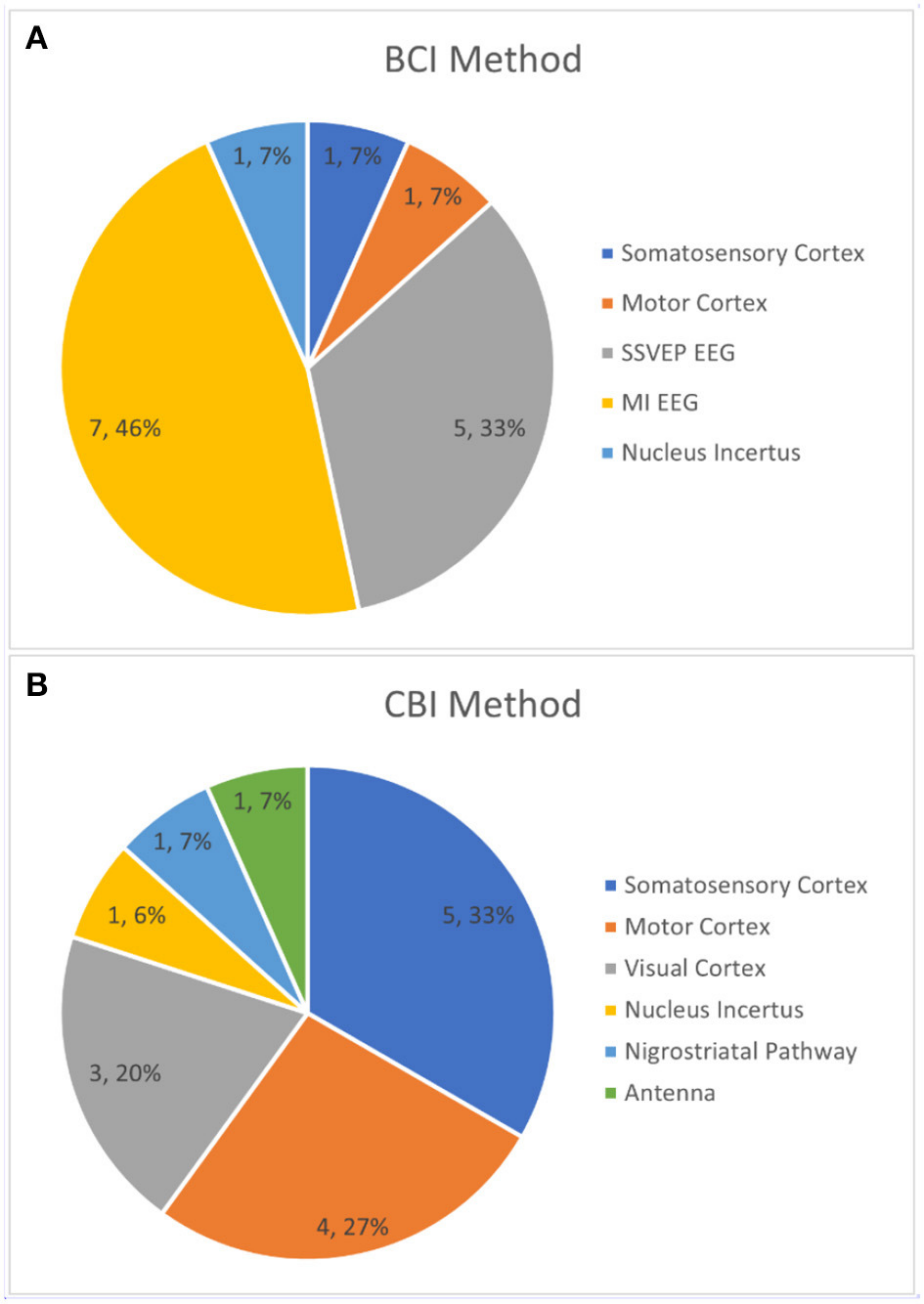
**(A)** The percentage of papers that target each region with their BCI. **(B)** The percentage of papers that target each region with their CBI.

The current status of B2BI research, based on the 15 selected papers, is shown in Figure 4 and Table 1. The most commonly used device for BCI is EEG (12 papers, 80% of the literature), specifically the method of targeting the motor cortex in a motor imagery (MI) task (7, 46.7%). ICM was the most common CBI technology employed (6, 40%), and most frequently stimulated the somatosensory cortex (5, 33.3%). The majority of papers utilized a direct unidirectional collaboration (11, 73.3%) and the large majority of papers utilized a 1:1 collaboration model (13, 86.7%).

**Table 1:** Summary of 15 brain-to-brain interfacing studies.

**BCI Device**	**BCI Method**	**CBI Device**	**CBI Method**	**Collaboration Type**	**Collaboration Model**	**References**
**(Neuroimaging)**		**(Stimulation)**				
EEG	MI	tMS	Visual Cortex	Direct Unidirectional	1:1 (Human:Human)	Grau et al., 2014
			Motor Cortex	Direct Unidirectional	1:1 (Human:Human)	Rao et al., 2014, Rajesh et al., 2020
				Indirect Bidirectional	1:1 (Human:Human)	Mashat et al., 2017
		ICM	Somatosensory Cortex	Direct Unidirectional	1:1 (Human:Rodent)	Yu et al., 2014, Zhang et al., 2019
		tFUS	Somatosensory Cortex	Direct Unidirectional	1:1 (Human:Human)	Lee et al., 2017
	SSVEP	tMS	Visual Cortex	Indirect Bidirectional	1:1 (Human:Human)	Stocco et al., 2015
					N:1 (Human:Human)	Jiang et al., 2019
		ICM	Antenna	Direct Unidirectional	1:1 (Human:Cockroach)	Li and Zhang, 2016
			Nigrostriatal Pathway	Direct Unidirectional	1:1 (Human:Rodent)	Koo et al., 2017
		tFUS	Motor Cortex	Direct Unidirectional	1:1 (Human:Rodent)	Yoo et al., 2013
ICM	Motor Cortex	ICM	Somatosensory Cortex	Direct Unidirectional	1:1 (Rodent:Rodent)	Pais-Vieira et al., 2013
	Somatosensory Cortex	ICM	Somatosensory Cortex	Indirect Bidirectional	N:N (Rodent:Rodent)	Pais-Vieira et al., 2015
Optogenetics	Nucleus Incertus	Optogenetics	Nucleus Incertus	Direct Unidirectional	1:1 (Rodent:Rodent)	Lu et al., 2020

### BCI Methodology

In the 15 B2BI studies analyzed, a total of 3 different neuroimaging technologies were applied to record neural signals. EEG was used by 80% (12) of the studies, ICM was used by 13.3% (2), and optogenetics was used by 6.7% (1) of the studies. Every study that involved human subjects employed EEG for the BCI portion of their experiment while ICM was used as a BCI exclusively for rodent-to-rodent studies. Optogenetics was only used once (Lu et al., 2020), and that study was also a rodent-to-rodent experiment. This discrepancy can be ascribed to the high accuracy of invasive methods such as ICM and optogenetics, assuming that the subject is willing to undergo the operation. In human studies, non-invasive methods take precedence despite their lower accuracy most likely because of ease of access, application, and data analysis.

Of the studies that employed EEG, either motor imagery (MI) or steady state visually evoked potentials (SSVEP) were measured by the BCI. MI EEG was used as a simple method of generating a binary signal, based on event related desynchronization (ERD) between the left and right hemispheres during right and left hand/feet movements, in Grau et al. (2014), Rao et al. (2014), and Rajesh et al. (2020). MI EEG also functioned as a direct translation, where the imagined movement of a hand corresponded to a similar action on the receiving end (right hand movement begets right turn, etc.) in Yu et al. (2014), Mashat et al. (2017), Lee et al. (2017), and Zhang et al. (2019). SSVEP EEG was applied in similar ways, with Stocco et al. (2015) using the visually evoked potentials to create a simple binary signal (subject focusing on one flashing LED or another) while Li and Zhang (2016) and Koo et al. (2017) used flashing LEDs on the left and right of a screen that corresponded to left and right movement of the receiver.

As mentioned, invasive methods were used for all the studies involving rodent-to-rodent transmission of information. The higher accuracy of these methods allowed for more unique applications. Pais-Vieira et al. (2013), the first study to employ a direct brain-to-brain paradigm, measured motor cortex activation in rats via ICM. Pais-Vieira et al. (2015) later went on to measure somatosensory cortex activation in rats in their network of rodent brains also using ICM. Lu et al. (2020) used optogenetically modified rats to measure activation of the nucleus incertus; this activation was used as a gauge of locomotion speed.

### CBI Methodology

In total, four different CBI technologies were used in the selected B2BI literature. Used equally most frequent were ICM and tMS, each used by 40% (6) of the studies analyzed. Beyond those two, 13.3% (2) of studies used tFUS and 6.7% (1) used optogenetics. As with the BCI methodology, CBI methodology is heavily dependent on the task. In terms of ICM applications, Yu et al. (2014) and Zhang et al. directly stimulated the somatosensory cortex to steer the movement of trained rats. At the same time, the somatosensory cortex has been stimulated by ICM for far more complicated tasks, such as rodent behavioral synchronization on a series of tasks (Pais-Vieira et al., 2013) and the creation of a biological neural network of rodent brains to classify stimulus and even forecast weather (Pais-Vieira et al., 2015). ICM was also used to steer a cockroach through a maze via antenna stimulation (Li and Zhang, 2016) and manipulate rat movement through a maze via nigrostriatal pathway stimulation (Koo et al., 2017).

TMS forms the bulk of CBI methodology with human subjects. Phosphenes, or tMS stimulation of the visual cortex to produce artifacts in a person's field of view, were used as a visual indicator of a binary choice (1 or 0, yes or no, left or right) in Grau et al. (2014), Stocco et al. (2015), and Jiang et al. (2019). The remainder of applications of tMS were for stimulating the human motor cortex either to press a button (Rao et al., 2014; Rajesh et al., 2020) or directly move a subject's limb (Mashat et al., 2017). Though less common, tFUS is also capable of non-invasively modulating neural activity. In B2BI literature, it has been used in humans targeting the somatosensory cortex to produce tactile sensations in the hands (Lee et al., 2017) and in rodents targeting the motor cortex to produce tail movements (Yoo et al., 2013). Lastly, as with their BCI, Lu et al. (2020) used optogenetically modified rodents in the CBI portion of their study. The rodent's nucleus incertus was hit with light to produce activation, allowing control of locomotive speed in the rat.

### Collaboration Type

**Figure 5:**
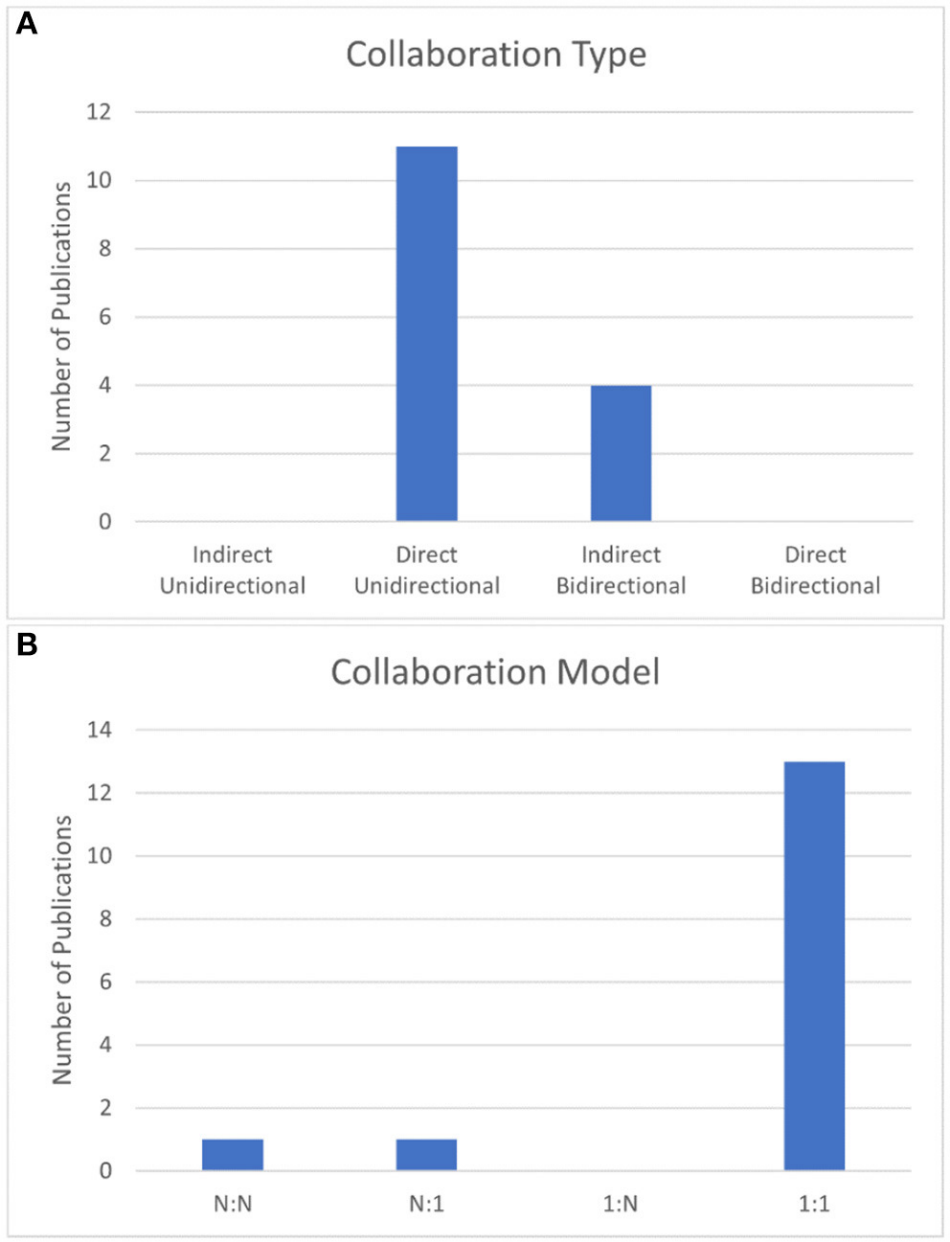
**(A)** Number of papers that employ each collaboration type. **(B)** Number of papers that employ each collaboration model.

As stated previously in this paper, B2BI designs can be broken down into four categories. These categories, and the percentage of selected B2BI papers that fall into each of them, are shown in Figure 5. Indirect unidirectional involves the transfer of information in only one direction between two brains, however the information transfer is not done through a neuromodulatory device. While the classification of indirect unidirectional exists, these studies are more akin to hyperscanning literature and are not classified as true B2BI. The large majority of B2BI literature, 73.3% (11) of it, uses a direct unidirectional collaboration type; direct unidirectional designs transmit information in one direction between subjects using some form of direct neuromodulation (tMS, ICM, etc.). These papers can be seen in every category of BCI and CBI systems, as shown in Table 1.

Less common are the indirect bidirectional studies which transmit information in both directions between subjects. These are labeled as indirect as they transmit information in one direction via neuromodulation, however the return of information to the sender is done through indirect methods such as visual feedback (e.g., messages on a computer screen). This collaboration type was employed by 26.7% (4) of the selected articles (Pais-Vieira et al., 2015; Stocco et al., 2015; Mashat et al., 2017; Jiang et al., 2019). Stocco et al. (2015) and Jiang et al. (2019) used a computer screen to close the loop and make the system bidirectional, however this is not the only approach. Mashat et al. (2017) utilized functional electrical stimulation (FES) of the original sender's arm to close the loop and signal that information has been sent *back* from the receiver. Pais-Vieira et al. (2015) directly transmitted information between a network of rodent brains using ICM. We chose to classify this as indirect bidirectional as the network was not fully connected (not every rodent connected bidirectionally to every other), though we acknowledge that the networked structure of their experiment does stretch the bounds of our definition.

### Collaboration Model by Subject Type

The collaboration model employed by each study is an important descriptor of how information was sent in the system and who the information was sent to. 46.7% (7) studies transmitted information from human subject to human subject, shown again in Table 1. There were several studies that employed cross-species B2BIs, with 26.7% (4) of the studies transmitting information from human to rodent (Yoo et al., 2013; Yu et al., 2014; Koo et al., 2017; Zhang et al., 2019). Pais-Vieira et al. (2013, 2015) specifically worked with only rodent to rodent transmission, as did Lu et al. (2020), forming 20% (3) of the literature. One study, Li and Zhang (2016) transmitted information from a human to a cockroach as well.

Collaboration model is not diverse in the selected literature. All but two studies, or 86.7% (13) of B2BI papers, use a 1:1 model. This means that information, regardless of directionality (unidirectional or bidirectional) is only transmitted between 2 subjects. This trend is broken only by Pais-Vieira et al. (2015) who employed a networked N:N model and Jiang et al. (2019) who employed a N:1 collaboration model.

### B2BI Definitions

The exact attributes of a B2BI system are not well-discussed. One third of the studies conducted in the field do not include a definition of brain-to-brain interfacing. Jiang et al. (2019) provides a comprehensive definition, stating that “brain-to-brain interfaces (BBIs) in humans are interfaces which combine neuroimaging and neurostimulation methods to extract and deliver information between brains, allowing direct brain-to-brain communication. A BBI extracts specific content from the neural signals of a ‘Sender’ brain, digitizes it, and delivers it to a ‘Receiver’ brain.” Alongside this paper, the large majority of studies, 8 out of the 10 that provide a definition, specify the existence of a BCI and CBI component. This aligns with the concept of neuromodulation being key in B2BI, and the requirement of a CBI in the information transfer (i.e., a direct rather than indirect).

In order to form as inclusive a definition as possible while still maintaining the critical attributes that make a B2BI system, we created a more standardized definition and provide with it a classification of B2BI devices into further categories. A B2BI is a system, composed of a BCI and CBI portion, that records one (or several) user's brain activity and uses it to directly modulate another (or several other) user's brain activity allowing information transfer between the two brains, with the CBI activation as a function of activity recorded by the BCI allowing the receiver to infer the sender's cognitive state. Reusing the terminology presented earlier, this definition further classifies B2BI systems as either direct unidirectional, indirect bidirectional, or direct bidirectional systems so long as they include a CBI that directly modulates a receiving brain at some point in the information transfer loop. Importantly, systems that employ only BCI devices or only neurostimulation (such as indirect unidirectional systems) are clearly excluded. Studies such as James (2011) and Maksimenko et al. (2018), mentioned previously, discuss and contribute to the realm of B2BI research, however they fail to demonstrate a system that meets the definition. In terms of further classification into categories relating to collaboration model, this definition includes systems involving more than just two subjects. Though very few studies include complex collaboration models at this time, we aim to include B2BI devices that exist now and that may exist in the future.

## Discussion

### B2BI Systems

Pertaining to the frequency with which each BCI device was used and how it was used, we found that 80% of studies selected used EEG. No other non-invasive BCI devices were reported, with the remainder of studies using either ICM or optogenetics in exclusively rodent models. These EEG based BCI systems employed either MI (46.7%) or SSVEP (33.3%). The invasive BCI methods on the other hand were able to target more specific locations (nucleus incertus, motor and somatosensory cortex itself). The application of non-invasive BCI methods for human subjects makes sense, as implanting electrodes in humans is not currently a commonplace procedure. EEG is the chosen option, most likely due to an abundance of literature in the field of BCI utilizing the technology, however EEG lacks the spatial resolution to identify complex brain activity due to the source localization problem. Of the most common EEG methods in the selected literature, MI has been limited to mostly binary information transfer potentially due to the difficulty of classifying more than two or three options at once. SSVEP, while being capable of more than two or three classes, is more akin to eye tracking than a true measure of user-generated brain activity.

Future research should explore other BCI hardware. Though far less practical in terms of size and ease of use, functional magnetic resonance imaging (fMRI) has shown potential for recognizing more abstract cognitive states and even emotions (Ruffini, 2016). A BCI method such as functional near-infrared spectroscopy (fNIRS) could potentially serve as a middle ground in terms of the spatial resolution of fMRI and the portability of EEG. With these new devices, the software portion of a B2BI will need to change as well. Current systems transmit mostly binary information and future applications of this technology, especially those applying more advanced neuroimaging methods, will require much greater throughput and more complicated handling of brain activity before the receiver is stimulated by the CBI.

In regards to analyzing the CBI devices used and where they targeted (RQ2), there is far less consensus. The majority of studies used either ICM (40%) or tMS (40%). These systems targeted mostly the somatosensory cortex (33.3%), with slightly fewer targeting the motor cortex (26.7%) and visual cortex (20%). Invasive CBI devices were exclusively used with rodent models while non-invasive CBI devices were used with both rodents and humans. Invasive CBI techniques are usually not preferred due to invasiveness-associated complications. Non-invasive CBI systems including transcranial direct/alternating current stimulation (tDCS/tACS), tMS, and tFUS have been actively investigated because of the clinical-friendly, non-invasive approach. Compared to other non-invasive neuromodulation methods, such as tDCS and tMS, tFUS is promising due to its excellent spatial selectivity and superior penetration depth (Lee et al., 2016). The mechanism of tFUS neuromodulation remains to be explored; low-intensity tFUS is theorized to exert acoustic radiation forces via the acoustic pressure waves which can interact with neuronal membrane to induce plasma membrane deformation, and affect mechano-sensitive ion channels, to modulate the activity of neurons (Tyler, 2012; Tyler et al., 2018). Other underlying mechanisms may involve the intramembrane cavitation induced sonophoresis (Krasovitski et al., 2011) and the thermal effect of ultrasound (Darrow et al., 2019). These unclear underlying mechanisms hinder scholars from choosing optimal ultrasound parameters to modulate the neural activity of the brain. In other words, different sonication settings of ultrasound frequency, pressure, intensity, waveform, could result in excitation or inhibition of neural activity, and cause various degrees of neurofeedback. Such a challenge may increase the difficulty of controlling CBI systems from the software perspective.

Besides the interaction between ultrasound and brain, another challenge for tFUS based CBI systems has to do with the interaction between ultrasound and skull. The acoustic attenuation and distortion caused by the skull has been treated as a barrier for transcranial ultrasound application for more than half a century (Hynynen and Clement, 2007). Over the past 20 years, scholars found that low-frequency ultrasound has less acoustic attenuation and distortion for transcranial ultrasound propagation. Also, the development of phased-array transducers makes it possible for transcranial ultrasound therapy by applying aberration correction (Clement and Hynynen, 2002). The low-intensity tFUS single-element transducer, rather than multi-element (phased-array) transducer, is the most common device for transmitting acoustic energy to the desired region through the skull due to its low cost and easy manipulation. However, in this case, it is hard to adjust the directivity and focal depth of ultrasound beam, which is a limitation of tFUS CBI systems to target specific areas inside the brain from the hardware perspective. To overcome such limitations, some promising methods have been proposed recently, which includes applying acoustic lens (Maimbourg et al., 2020) or holographic plates (Jiménez-Gambín et al., 2019) in front of the tFUS transducer to achieve adjustable acoustic beam steering and focusing. Furthermore, new applications of tFUS based CBI systems may be explored more. As an example, a sonogenetics approach can be used to stimulate specific neurons in the desired area of the brain (Ibsen et al., 2015).

Improvements and standardizations in the realm of defining these systems is also an important step, as the definitions provided in the literature are inconsistent. Studies focus too much on their application for transmitting motor information, as in Mashat et al. (2017), overly specify attributes such as wireless transmission, as in Rajesh et al. (2020), or only specify the inclusion of a BCI and CBI portion and very little else, as in Rao et al. (2014) and Li and Zhang (2016). More cohesion in the field as to what type of B2BI is being presented would allow for quicker communication of applications for these devices. Ideally, a more robust definition like the one provided here will aid and expedite discussion about this budding technology.

### Experiment Design

We found the design of B2BI systems in the collected literature to be lacking in diversity. As collaboration and communication are some of the core applications of B2BI, results in this area are key in demonstrating the potential of the technology. The bulk of literature followed a direct unidirectional design (73.3%) while no studies implemented a direct bidirectional design (neuromodulation on both the sender and receiver). In a similar fashion, almost every study employed a 1:1 collaboration model (86.7%). More complex models were very rare. The majority of 1:1 studies employed either a human:human model (46.7%) or a human:rodent model (26.7%). The frequency of these methods is understandable; the field is very young and unidirectional models must logically predate bidirectional models. Beyond that, the use of human-to-rodent and rodent-to-rodent models is also indicative of the precaution being taken in the realm of CBI safety with human subjects. High confidence in neuromodulation within human subjects is needed to expand into more complex designs, such as networked human brains in an N:N model resemblant of Pais-Vieira et al. (2015). Unidirectional models are also likely far more common as bidirectional models would double the cost of the device, making indirect bidirectional systems (utilizing peripheral nervous pathways for the response) more reasonable.

Future research needs to expand into more complex collaboration designs and test the capabilities of B2BI. Jiang et al. (2019) is the only study to employ a 2:1 collaboration model, where the receiver had to identify which sender was more reliable (due to the introduction of noise to a random receiver's signal). Multiple sender systems such as this more closely resemble the diversity of some real world applications, and investigation into 1:N collaboration models (with one sender broadcasting to a group of receivers) should follow suit. To see the true potential of collaboration in these devices, systems such as Pais-Vieira et al.'s (2015) N:N model warrant further exploration as well. Though uses in the domain of human subjects may not be employed as biological neural networks, a network of collaborating brains exchanging information to and fro could hold useful applications in cases requiring complex teamwork. All of these future directions require investigation into direct bidirectional systems, something we have not seen yet in B2BI literature. Transmission of information directly between brains, both to and from each subject, is necessary to explore complex applications of B2BI technology.

Unfortunately, and similar to the procedure used with existing CBI systems such as tMS, tFUS neuromodulation usually requires patients to get CT or MR scans first to provide researchers the skull morphology. Then, based on such information and through the assistance of a neuronavigation system, an appropriate ultrasound beam with designed acoustic parameters can be generated and transmitted into the desired region of the brain for a given period of time. Given the two limitations of tFUS-based CBI systems mentioned in B2BI Systems, how to monitor and evaluate the ultrasound beam inside the desired region of the brain would be a key to increase the success rate, and decrease the risk of CBI experiments. Future work should include mention of imaging guidance, temperature and neural response monitoring for the sake of safety and effectiveness, information that is lacking in some tMS and tFUS B2BI studies. Since acoustic radiation force plays a crucial role for ultrasound neuromodulation, magnetic resonance-acoustic radiation force imaging (MR-ARFI) method can be used to specify the location, and quantify the magnitude of ultrasound beam inside the brain (Phipps et al., 2019). Furthermore, MR-thermometry is a useful tool to show the temperature rise in the sonication area of the brain (Ozenne et al., 2020). With these methods, it is expected to ensure the operational safety of ultrasound neuromodulation for clinical applications. Meanwhile, functional magnetic resonance imaging (fMRI) has shown its effectiveness for measuring neural activity of the brain (Beisteiner et al., 2020), and could help researchers choose optimal sonication parameters for future CBI and B2BI studies.

### Applications

Applications of B2BI range from rehabilitation and treatment to communication, collaboration and synchronization. After an injury that is potentially treatable by brain stimulation, such as a stroke, activation motor regions of the brain can help the patient recover faster. Activation of motor cortex via brain stimulation such as tMS can help promote neuroplasticity and a relearning of lost motor ability (Neren et al., 2016). A physical therapist could, through a B2BI, issue motor commands to a patient during rehabilitation to assist in recreating lost pathways in their brain. Something similar to this was done by Mashat (2017) using functional electrical stimulation (FES) of the arm; the next logical step up from FES would be direct neural stimulation rather than muscular. This application of B2BI could potentially expedite rehabilitation of post-stroke patients through neuroplasticity. Beyond the professional-patient relationship, B2BI has significant future applications in communication and collaboration. In the first B2BI study, Pais-Vieira et al. (2013) demonstrated that rodents connected to a B2BI could learn to synchronize their behavior without any peripheral nervous system cues (such as sight of the other rat). Behavioral synchronization such as this could be very advantageous in a workplace where it is important for workers to move with each other during a complex task. Adding to this a networked, or at least >1:1, collaboration model could result in a team of workers moving as a collective unit while completing a potentially hazardous task. Stocco et al. (2015) posits that B2BI could find application in communication between users when traditional verbal communication falls short, such as in users with Broca's aphasia or even different native languages. These applications are supported by the relatively small body of literature we have to date, but as research and technology progresses futuristic applications become less futuristic.

As BCI technology becomes more capable of recording nuanced brain activity and CBI technology more precise at stimulating the brain, it becomes more possible to transmit complex information between B2BI users. Future B2BI devices could transmit abstract thoughts, memories, or emotions from user to user, things that are often quite difficult to convey to other humans through conventional means. As the body of research continues to grow, so do the possibilities and applications of this technology.

## Conclusions

We systematically identified, critically appraised, and synthesized 15 relevant studies on brain-to-brain interfaces for information transmission between brains. These studies, all published after 2013, fit the pre-specified inclusion and eligibility criteria. We used an explicit systematic method, the preferred reporting items for systematic reviews and meta-analyses (PRISMA) to address 4 specific questions regarding BCI methodology (RQ1), CBI methodology (RQ2), collaboration type (RQ3), and collaboration model by subject type (RQ4). We also present a wide-encompassing definition of a brain-to-brain interface to simplify later reviews and classification.

Future challenges and directions for B2BI research demonstrated in this review include:

- The lack of consensus on CBI methodology, indicating the existence of benefits and drawbacks to each region of the brain that may have been chosen.
- Very little diversity in CBI technology used. The large majority of studies used either tMS or ICM. In terms of non-invasive neuromodulation, other devices such as tFUS and transcranial direct/alternating current stimulation (tDCS/tACS) could allow novel applications of the technology in future studies (Rao et al., 2014).
- Only a few studies that employ complicated collaboration designs showed that few researchers have looked to stretch the limits of what B2BI may be capable of.
- No direct bidirectional collaboration types, only studies using peripheral nervous pathways (visual feedback). Future research into direct bidirectional systems could allow B2BI communication while performing more complicated tasks.

This systematic review is unfortunately limited in several ways due specifically to the limited number of publications. The PRISMA system could not be employed in its entirety as certain measures, like the PICOS statement, were too limiting for the small number of papers. Rather than filter the papers analyzed by metrics such as participants and interventions, specific research questions were listed in detail for analyses and as many experimental papers were selected as possible. The small number of papers targeted also poses possible problems regarding bias within and across studies. In order to most thoroughly present the state of B2BI research though, all 15 studies were included and analyzed. All limitations considered, this systematic review, based on the findings of documented, transparent, and reproducible searches, should help build cumulative knowledge and guide future research regarding direct communication between brains via B2BIs. The summarized findings herein will hopefully help facilitate new discoveries and experimentation to push the boundaries of brain-to-brain interfacing.

## Data Availability Statement

The original contributions presented in the study are included in the article/supplementary material, further inquiries can be directed to the corresponding author/s.

## Author Contributions

All authors listed have made a substantial, direct and intellectual contribution to the work, and approved it for publication.

## Conflict of Interest

The authors declare that the researchs was conducted in the absence of any commercial or financial relationships that could be construed as a potential conflict of interest. The handling Editor declared a past co-authorship with one of the authors CN.
